# London Correspondence

**Published:** 1867-08

**Authors:** Lunsford P. Yandell

**Affiliations:** London


### EDITORIAL AND MISCELLANEOUS·

LONDON, July 3, 1867.

DR. J. M. JOHNSON.

*My Dear Sir*,—Of the many very clever young medical men in London, Dr. Tilbury Fox is the most eminent. He is one of the editors of the London *Lancet*, and is destined to attain great distinction, being yet only a little over thirty years old, a man of a high order of intellect and of unbounded energy. His devotion to medicine is perfect, and his attainments already varied and extensive. I owe him many obligations for opportunities enjoyed for prosecuting my researches here to the greatest advantage, and take pleasure in making the acknowledgment for the benefit of my young countrymen, who may hereafter come to this metropolis to improve their knowledge of medicine. I can pledge them a kindly reception from this affable gentleman. I have been particular to give you his name in full, because there are nearly as many Foxes in England as were once maltreated by Samson; but, although there are two or three prominent men in the profession in London by that name, there is but one Dr. Tilbury Fox. He devotes himself specially to diseases of the skin, upon which subject he has written two books, besides many able articles in the *Lancet.*

Through the civilty of Dr. Fox, I had an opportunity a few evening's since, of being present at a meeting of the Epidemiological Society; and I have an engagement to attend with him a doctor's picnic in the country, where, he assures me, I shall see something of English country life, and see the doctors on a frolic. This meeting is said to have been the most interesting held by the society during the whole medical session. Papers were read by Dr. Lyons and Dr. Mapother, of Dublin, and Marston, of the Army Medical Department, on an epidemic which is, at present, attracting much notice in this country. It has been variously styled "the black death," "the black fever," "spotted fever," and "cerebro-spinal meningitis," to give no other names, and the character and treatment of the epidemic remain unsolved problems. The chief symptoms point to sudden blood poisoning, which is followed by inflammation of the brain and its meninges, with liquefaction of the blood. The vital powers are suddenly depressed, delirium and loss of consciousness supervene, purple blotches occur over the skin, spasm of the muscles comes on, followed by a livid countenance, coldness of the body, and death at an early date.

Dr. Lyons, when the epidemic appeared in Dublin, a year ago, pronounced it a new thing, and felt that he had a novel affair to deal with. Some cases have lately occurred in London, and the profession has been stirred up to a very energetic investigation of the malady. The discussion which followed the three able papers at the Epidemiological Society, was highly interesting. The fact that the disease had appeared in the United States was adverted to, and many statistics connected with its spread in Ireland were given. Its mortality has everywhere been appalling. Of twenty-five attacks in one region, nineteen were fatal. In all Ireland, 43 have died out of 76, or more than 50 per cent. There is no reason to believe the disease contagious. It manifests a partiality for children; and it was stated in the course of the evening, that the "purples" in hogs have, in all parts of the world, been coincident with the disease.

The idea has been repeatedly advanced, that this disease has a close relation to scurvy; but the discussion showed that there was no solid ground for the opinion. It was also shown that it differs essentially from typhus fever, with which it has been confounded. Dr. Jenner, the President of the society, pointed out the differences between the two diseases in a conclusive manner. Typhus seldom attacks children, and when it does, can hardly be said to be a fatal affection. In typhus, the progress is slow, and inflammation of the brain is seldom found; while the suddenness of the seizure, and the early occurrence of "spots," and speedy dissolution, serve to distinguish the new disease from the old.

The President, after the admirable papers had been read and commented upon by the numerous members, remarked that there was "a medical gentleman present from America, he understood, and hoped he would favor the Society with his knowledge on the subject of this disease." After so courteous an introduction, I could not decline making some remarks, and stated, among other things, some reasons why I thought the disease had nothing scorbutic in it. I referred to the fact, that during the civil war, when there was great scarcity of vegetable food, and insufficient food of all sorts in the Confederacy, much of the time, and coincidently with this, much scurvy, no spotted fever occurred. At Corinth, for example, where we lost thousands of men from scorbutic diarrhœa and dysentery, and fever diagnosed typhoid, we had no spotted fever. In regard to the epizoötic diseases of the lower animals, which had been referred to in the discussion, I related a number of facts in which the members appeared interested. I allude to the "purples" or cholera in the hog, "blind staggers," and the epidemics which have been so destructive of poultry in many regions of our country. The wide and fatal prevalence of "hog cholera," I stated, was one of the causes of the great scarcity of animal food in the South during the war; our people having been accustomed to rely chiefly upon the flesh of the hog for subsistence. Nothing can exceed the urbanity of our professional brethren in London; and the consideration with which they received my unpremeditated remarks, was peculiarly gratifying to a stranger. I am sorry that I cannot report anything more definite with regard to the origin or the nature of spotted fever, and can give absolutely nothing with respect to its treatment that promises better results in future than those which have attended past efforts in that direction.

The *Sphygmograph*—an instrument by which the arterial pulse is made to register itself upon paper—is attracting a great deal of professional attention in this city. Two exceedingly interesting lectures were delivered a short time since in the theatre of the Royal College of Physicians, by Dr. Austie, one of the great medical men of London, on the prognosis and treatment of certain acute diseases, with special reference to the indications afforded by this instrument. Its use as an aid to prognosis in acute diseases, was explained; and it was also shown that from its record of the pulse, valuable indications as to the use of alcohol might be derived. If the stimulant renders the pulse more dicrotos and more rapid, it is injurious; if it makes it slower and less dicrotos, it does good. These important lectures will soon be published, and will attract unusual attention.

Dr. Prothroe Smith has been industriously investigating the powers of a new anæsthetic, and has lately delivered some lectures on the subject, embodying a number of highly interesting facts. He has administered the *tetra-chloride of carbon*, with happy results in a variety of disorders. It is inhaled to the extent of a drachm, at intervals, and though the anæsthetic effect is transient, he has seen relief follow its administration in headache, dysmenorrhœa, chronic metritis, and hay-fever. He has also used it in labor, and the following is a summary of his experience in the various cases to which he has applied it. The tetra-chloride of carbon will be found useful in removing pain in headache, dysmenorrhœa, tic doloreux, toothache, etc., and will be a valuable and safe means of mitigating the pains of labor, without, apparently hindering the natural efforts. In some cases of labor, it induces a quiet sleep, and removes for a time the efforts of exhaustion of the nervous system. In hay-fever, the distressing local irritation is allayed by its vapor inhaled a short time. In many respects, Dr. Smith deems it preferable to chloroform—quite as safe, pleasanter to inhale, and producing the effect desired in a smaller quantity.

Insanity is said to be on the increase in some countries of Europe; and especially the form of madness called *folly* or *title-madness.* In 1861 there were only 184 cases in Prussia; in 1866 there were 236. Instances of ambitious folly have predominated. Among the patients struck with this form of maniæ, there were five false Napoleons (III.), two false Popes, one President Lincoln, three Grand Dukes of Holstein, three Emperors of Mexico, eight Kings of Prussia, two Emperors of Austria, one Count Bismarck, and ten Emperors of Germany.

The lectures of Dr. Anstie, of which I have spoken, were attended by the leading practitioners of London, amongst whom I recognized Sir Thomas Watson, author of the very popular work on the Practice of Medicine. An eminent medical man belonging to "young medicine," said to me, speaking of Sir Thomas, "he is a darling old gentleman; he is always attentive to young men in the profession, and encourages them and helps them along. He has retired from practice, and doesn't care for this thing, but has come to hear the lectures out of compliment to Dr. Anstie. Sir Thomas Watson is a stout old gentleman, with mild, benignant face, immense head, sparingly fringed with white hair, and the whole language of his body tells you plainly that he *is* "a darling old gentleman."

Speaking of this great author on Practice, reminds me of a compliment paid by Dr. Anstie, in his lectures, to our friend, Dr. Austin Flint. He spoke of him as "that excessively clever American physician;" of course, using "clever"

in the English sense. I find that, not only in Dr. Anstie's opinion, but with the profession generally in England, Dr. Flint's book on Practice ranks infinitely higher than that of Dr. Tanner.

Of the sphygmograph, without attempting a full description of it, it may, perhaps, be interesting to say that it is a small brass instrument, eight or ten inches long, which is fitted to the wrist by elastic bands. A lever on top of the instrument is moved by the pulsations of the artery, and on the free end of this lever is a pen, which writes upon a piece of smoked glass, moved along past the pen by machinery. Sometimes ink and paper are used instead of the smoked glass. The lines drawn by the pen vary in irregularity with the variations of the pulse.

One is astonished at some of the contrarieties of medical opinion in this great capital. For instance, there are accouchers here, of good standing, who still oppose the use of chloroform in labor, on scriptural grounds. They are regarded, however, as fossils in the profession, and are looked upon as curiosities, not authorities.

Mr. Lane and Mr. Gascoine, who were appointed by the Royal Medical and Chirurgical Society, to investigate syphilization, have made a report which kills that most unpromising operation dead. Henceforth, syphilization in England will only be named to be denounced or laughed at. *Hydrophobization*, or *rattlesnakization* would be fully as popular, if some fanciful individual should propose to prevent injury from mad-dogs, or snake-bites, by inoculation.

LUNSFORD P. YANDELL, Jr.

